# Screening for Pre-Frailty Using Phase Angle Derived from Bioelectrical Impedance Analysis in Community-Dwelling Older Adults

**DOI:** 10.3390/geriatrics11020049

**Published:** 2026-04-20

**Authors:** Masayuki Hoshi, Tomoka Ogata, Maaya Chiguchi, Ayane Nakamaru, Tatsuya Nakanowatari, Akihiko Asao, Natsumi Kimura, Maki Ogasawara, Yuko Horikoshi, Rie Sakuraba-Hirata, Akiomi Yoshihisa, Hiroshi Hayashi, Toshimasa Sone, Yoshitaka Shiba

**Affiliations:** 1 Department of Physical Therapy, School of Health Sciences, Fukushima Medical University, Fukushima 960-8516, Japan; nakanowa@fmu.ac.jp (T.N.); y-shiba@fmu.ac.jp (Y.S.); 2 Department of Rehabilitation, Ohara General Hospital, Fukushima 960-8611, Japan; 3 Department of Rehabilitation, Japanese Red Cross Fukushima Hospital, Fukushima 960-8530, Japan; 4 Department of Rehabilitation, Takeda General Hospital, Fukushima 965-8585, Japan; 5 Department of Occupational Therapy, School of Health Sciences, Fukushima Medical University, Fukushima 960-8516, Japan; a-asao@fmu.ac.jp (A.A.); k-natsu@fmu.ac.jp (N.K.); oga-maki@fmu.ac.jp (M.O.); hhayashi@fmu.ac.jp (H.H.); tsone@fmu.ac.jp (T.S.); 6 Department of Clinical Laboratory Sciences, School of Health Sciences, Fukushima Medical University, Fukushima 960-8516, Japan; ykokubun@fmu.ac.jp (Y.H.); hrt@fmu.ac.jp (R.S.-H.); yoshihis@fmu.ac.jp (A.Y.)

**Keywords:** phase angle, pre-frailty, bioelectrical impedance analysis, older adults, screening

## Abstract

**Background/Objectives:** To evaluate the utility of phase angle (PhA) derived from bioelectrical impedance analysis as a screening indicator for pre-frailty in community-dwelling older adults. **Methods:** This cross-sectional study included 171 participants (36 men and 135 women) in Japan in 2023. PhA at 50 kHz was measured using bioelectrical impedance analysis and evaluated as a potential screening indicator for pre-frailty. Assessments included body composition, physical function tests (maximum walking speed, Timed Up and Go (TUG), grip strength, knee extension strength, and one-leg stance time with eyes open), cognitive function (MoCA-J), and the Motor Fitness Scale (MFS), a questionnaire assessing physical function, along with the Kihon Checklist (KCL). Frailty status was defined using KCL scores (4–7: pre-frailty; ≥8: frailty), and participants were classified into robust and pre-frail/frail groups. **Results:** PhA was significantly correlated with physical function measures, including grip strength (r = 0.54, *p* < 0.01), MFS (r = 0.36, *p* < 0.01), maximum walking speed (r = 0.20, *p* < 0.05), knee extension strength (r = 0.16, *p* < 0.05), and TUG (r = −0.17, *p* < 0.05). In women, logistic regression analysis showed that PhA was independently associated with pre-frailty (age-adjusted odds ratio: 2.38; 95% CI: 1.08–5.23; *p* < 0.05). ROC analysis yielded an area under the curve of 0.65 (95% CI: 0.56–0.74), indicating modest discriminative ability. Age-adjusted cutoff values of PhA were 4.19° and 4.74°, corresponding to points prioritizing sensitivity and specificity, respectively. **Conclusions:** PhA is associated with physical function and may serve as a simple, non-invasive indicator for identifying pre-frailty in community settings. However, given its modest discriminative ability, PhA alone may not be sufficient as a standalone screening tool and should be used in combination with other clinical indicators for clinical application.

## 1. Introduction

Aging of the population is associated with an increased risk of frailty. Frailty is an intermediate state between a robust condition and a state requiring long-term care. It is characterized by increased vulnerability to stressors and a higher risk of adverse outcomes, including functional decline, dependency, hospitalization, and mortality [1]. Frailty encompasses physical, psychological, and social domains, which are closely interrelated [1,2]. Numerous studies have demonstrated that physical frailty increases the risk of falls, hospitalization, and mortality [1,3,4,5]. Although frailty is potentially reversible, recovery may be difficult in the presence of multiple age-related comorbidities. Therefore, early identification of frailty, particularly at the pre-frailty stage, is essential for preventing its progression. Pre-frailty is associated with declines in lower limb function, gait performance, and balance during activities of daily living (ADL) [6], highlighting the importance of early preventive interventions.

Phase angle (PhA), derived from bioelectrical impedance analysis (BIA), reflects cellular integrity and overall nutritional status and has been recognized as a useful clinical indicator [7]. It has been applied in various fields, including the assessment of disease severity and the prediction of postoperative complications, disability, and mortality [8]. Higher PhA values indicate better cell membrane integrity, with typical values ranging from 5° to 7° in healthy adults; women generally exhibit lower values than men [9,10]. Previous studies have shown that lower PhA values are associated with an increased risk of disability [11] as well as a higher risk of falls and fear of falling [12]. In addition, PhA has been widely used as an indicator of nutritional status and risk of malnutrition, reflecting alterations in cellular health. Despite these findings, few studies have specifically examined the relationship between PhA and pre-frailty in community-dwelling older adults.

This study aimed to evaluate the utility of PhA derived from bioelectrical impedance analysis as a screening indicator for pre-frailty in community-dwelling older adults.

## 2. Materials and Methods

### 2.1. Participants

**Figure 1:**
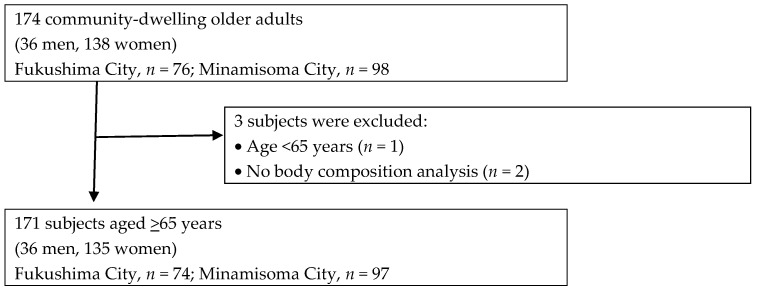
Flowchart of participant selection for the study cohort.

The subjects were community-dwelling older adults in two areas in Fukushima Pre-fecture (Fukushima City, Minamisoma City) who were able to walk to the venue. They were recruited through administrative publications such as public information magazines. Of the 174 participants in the project, 171 individuals (36 men, 135 women) were included in the present analysis, after excluding those under 65 years of age and those who did not undergo body composition analysis. A flowchart of participant inclusion is shown in Figure 1.

### 2.2. Methods

The assessment items included physical function evaluations, such as: grip strength, knee extension strength, one-leg stance time with eyes open, 10 m maximum walking time (maximum walking speed) and Timed Up and Go (TUG) test; cognitive function tests, including: Japanese version of Montreal Cognitive Assessment (MoCA-J); and various questionnaires, such as: Ministry of Health, Labour and Welfare Kihon Checklist (KCL) and Motor Fitness Scale (MFS) [13,14]. All functional tests were conducted according to standardized procedures by trained examiners to ensure measurement consistency. These assessments have been widely used in previous studies and have demonstrated good reliability and validity in older adults.

#### 2.2.1. Body Composition Analysis

Body composition analysis was performed using an eight-point contact electrode method with a bioelectrical impedance analyzer (InBody S10; InBody Co., Ltd., Tokyo, Japan). Measurements were conducted with participants in the supine position on a bed, with electrodes attached to both thumbs, middle fingers, and ankles. Each measurement required approximately 100 s. The measured parameters included PhA at 50 kHz obtained by bioelectrical impedance analysis and the skeletal muscle mass index (SMI). The InBody S10 determines impedance by measuring resistance and reactance using a multi-frequency current, and the device has been reported to provide stable and reproducible measurements under standardized conditions.

#### 2.2.2. Grip Strength

Patients were asked to stand with their feet slightly apart, and to hold the grip dynamometer (Digital Grip Dynamometer T.K.K.5401, SANKA Co., Ltd., Niigata, Japan) without touching it to their body. Grip width was adjusted so that the proximal interphalangeal joints were vertical when gripping. Measurements were performed twice, and the maximum value in each measurement was evaluated. As a rule, measurements were taken in the dominant hand.

#### 2.2.3. Knee Extension Strength

The subject crossed their arms in front of their chest while seated on a chair with the knee joint flexed to 90°, and a handheld dynamometer (μTasF-1, Anima Co., Ltd., Tokyo, Japan) sensor was placed directly above the ankle joint of the dominant leg for measurement. The isometric knee extension test was performed to measure maximum muscle strength with 5 s isometric contraction of the quadriceps muscle at the knee joint, with two measurements being performed. Maximum muscle force values (N) were calculated and multiplied by the distance from the knee joint cavity to the sensor center to measure torque (Nm). Subsequently, muscle strength (Nm/kg) was calculated by dividing the aforementioned calculated values by the subject’s body weight. Although measurements were primarily performed on the dominant leg, the non-dominant leg was used if pain, fatigue, or other impediments were present. Measurements were performed twice, and the higher of the two values was used in assessments.

#### 2.2.4. One-Leg Stance Time with Eyes Open

The one-legged stance time with eyes open measured the duration of standing with one foot lifted off the floor. The measurement ended when the subject changed the position of the supporting leg, when the raised leg touched the floor, or when any part of the body touched the wall or other support surface. Measurements were taken in the dominant leg as a rule, but were performed with the non-dominant leg if pain, fatigue, or other impediments were present. Measurements were conducted with the participants eyes open, and the upper limit was set at 60 s.

#### 2.2.5. Maximum Walking Speed

Two tape markers were applied at a distance of 10 m apart on a flat floor. Additional tape was applied 2 m in front of and behind each main tape mark, creating a total distance of 14 m. The time required to walk 10 m was measured using a stopwatch. Before the measurement began, subjects waited in a stationary standing position at the starting point of the preliminary path, and were uniformly instructed to “walk as fast as you can”. Measurement of walk timing commenced when the leading foot touched or crossed the 10 m starting line and ended when the leading foot touched or crossed the 10 m finish line.

#### 2.2.6. TUG

For the TUG measurement, a smartphone app was used to record the TUG time. The Hacaro Instrumented Timed Up and Go app (hereafter, iTUG) (Digital Standard Co., Ltd., Osaka, Japan) was employed to measure the time taken to go from the seated position to standing up, walking to a marker 3 m away, turning around the marker, and returning to sit down on the chair. The instruction given was: “Walk as fast as possible without running”. The direction of turning was at the subject’s discretion.

#### 2.2.7. Cognitive Function

The MoCA-J was used to assess cognitive function. The MoCA-J is a 30-point scale that evaluates multiple domains of cognitive function (visuospatial, executive, attention, language and orientation) in approximately 10 min, with a score of 25 or lower defining mild cognitive impairment (MCI) [15].

#### 2.2.8. KCL

The KCL is a self-administered questionnaire consisting of 25 items regarding living conditions and physical/mental function, answered as “Yes” or “No.” It comprises seven domains: five items assessing activities of daily living, five items assessing musculoskeletal function, two items assessing malnutrition, three items assessing cognitive function, and five items assessing depressive mood. A score of 4–7 points is classified as pre-frailty, while 8 points or higher is considered to indicate frailty [1].

#### 2.2.9. MFS

The MFS [13,14] is a questionnaire consisting of 14 items that allows for a simple and safe assessment of motor abilities, such as mobility, muscle strength and balance. Participants respond with ‘yes’ or ‘no’ answers, with “yes” receiving 1 point and “no” receiving 0 points, for a total possible score of 14 points.

### 2.3. Statistical Analysis

For statistical analysis, subjects were classified into pre-frail/frail (KCL score ≥ 4) and robust groups (KCL score 0–3) based on the KCL criteria. The normality of data distribution was assessed using the Shapiro–Wilk test. Variables with a normal distribution are presented as mean ± standard deviation (SD), while non-normally distributed variables are presented as median and interquartile range (IQR). The Mann–Whitney U test (nonparametric *t*-test) was used to compare basic attributes and measurement results between the two groups. Spearman’s rank correlation coefficient was employed to examine correlations between body composition analysis, physical function assessments, and questionnaire responses. Logistic regression analysis was performed to examine factors associated with frailty. Odds ratios (ORs) and 95% confidence intervals (CIs) were calculated. Statistical significance was set at *p* < 0.05. Additionally, logistic regression analysis and cutoff value evaluation to examine the relationship between pre-frailty and PhA were performed only for women. Receiver Operating Characteristic (ROC) curves and Area Under the Curve (AUC) were used to evaluate cutoff values. Effect sizes were calculated to complement *p*-values and are presented as correlation coefficients (r) or Cohen’s d, as appropriate. Receiver operating characteristic (ROC) curve analysis was performed to determine cutoff values, and the area under the curve (AUC) with 95% confidence intervals (CIs) was calculated. The significance level was set at 5%. IBM SPSS Statistics 29.0 was used for statistical analysis.

### 2.4. Ethics Approval and Consent to Participate

The research protocol was reviewed and approved by Fukushima Medical University (approval No.: General 2022-123; 1 September 2025). The informed consent process was conducted by the organizing municipalities using an opt-in procedure, and written informed consent was obtained from all participants. This study was conducted in accordance with the principles of the Declaration of Helsinki.

## 3. Results

### 3.1. Basic Characteristics by Sex of the Study Participants

Table 1 shows the basic attributes and basic results of statistical analyses for each measurement item.

### 3.2. Sex-Related Comparison of Pre-Frail/Frsil and Robust Groups Based on KCL Scores

The results of comparison of each measurement item between groups divided into pre-frail/frail and robust groups based on KCL scores are shown in Table 2. In men, comparison of the pre-frail/frail and robust groups revealed significant differences in the KCL questionnaire total score, MFS total score, knee extension strength, one-leg stand time with eyes open. While no significant difference was found in PhA, the pre-frail/frail group scored lower than the robust group. For women, comparison of the pre-frail/frail and robust groups revealed significant differences in the KCL questionnaire total score, MFS total score, MoCA-J, knee extension strength, TUG, maximum walking speed, and PhA in the physical function assessment.

### 3.3. Scatter Plots Showing the Correlation Between Body Composition-Derived PhA and Motor Function

**Figure 2:**
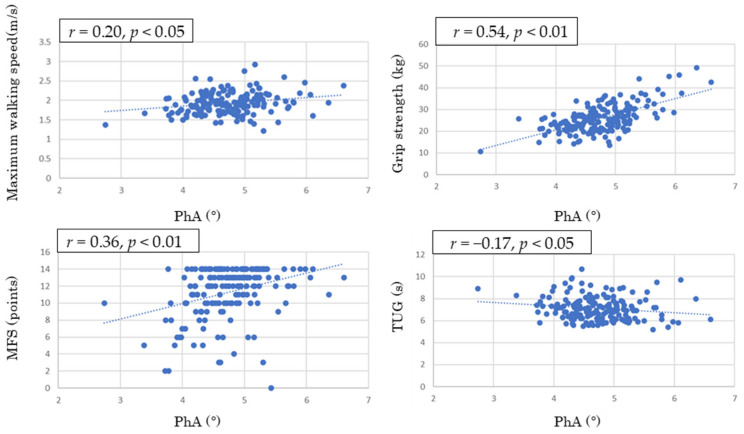
Scatter plot showing the correlation between body composition-derived PhA and motor function. Notes: Values are presented as Spearman’s correlation coefficients (r). The dashed line represents the fitted regression line indicating the relationship between variables. PhA, phase angle; MFS, Motor Fitness Scale; TUG, Timed Up and Go.

Figure 2 shows correlation plots between PhA and questionnaire scores and physical function assessments. The corresponding correlation coefficients are also summarized in Table 3. The correlation coefficient between PhA and the MFS total score was 0.36 (*p* < 0.01), and that between PhA and grip strength was 0.54 (*p* < 0.01), indicating moderate positive correlations. Furthermore, the correlation coefficient between PhA and knee extension strength was 0.16 (*p* < 0.05), and that between PhA and maximum walking speed was 0.20 (*p* < 0.05), indicating weak positive correlations. The correlation coefficient between PhA and TUG was −0.17 (*p* < 0.05), indicating a weak negative correlation.

### 3.4. Logistic Regression Analysis Model with Pre-Frailty Status as the Dependent Variable (Women Only)

Table 4 shows the results of multivariate logistic regression analysis of factors associated with frailty in older women. In the multivariate logistic regression analysis including phase angle and age, phase angle was significantly associated with pre-frailty. Phase angle showed an OR of 2.38 (95% CI 1.08–5.23, *p* < 0.05), and age showed an OR of 0.96 (95% CI 0.89–1.03, *p* = 0.22). In men, no significant association was found between PhA and the presence or absence of pre-frailty in the logistic regression model.

### 3.5. Use of PhA for Screening for Pre-Frailty in Older Women

**Figure 3:**
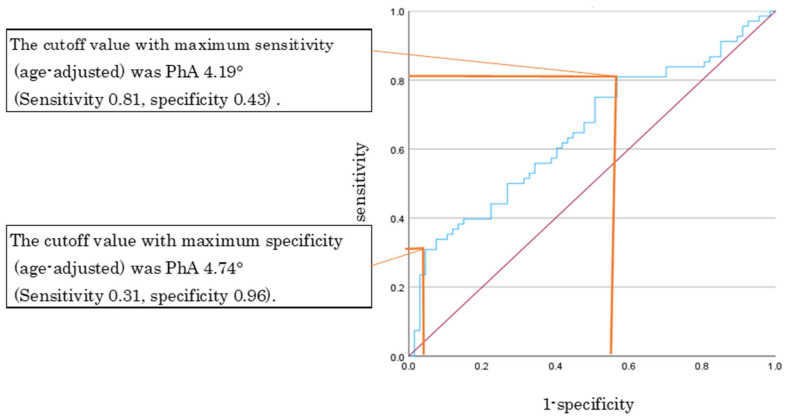
Use of PhA (50 kHz) for screening for pre-frailty in older women. Notes: The blue line represents the ROC curve. The red line indicates the reference line (AUC = 0.5). The orange point indicates the optimal cut-off value based on the maximum Youden index (maximum sensitivity and specificity).

Figure 3 shows the results of use of PhA (50 kHz) for screening for pre-frailty in older women. ROC analysis yielded an AUC of 0.65 (*p* < 0.05, 95% CI: 0.56–0.74). The cutoff values (after age adjustment) that maximized both sensitivity and specificity were a PhA of 4.19° (sensitivity 0.81, specificity 0.43) and 4.74° (sensitivity 0.31, specificity 0.96), respectively.

## 4. Discussion

This study examined pre-frailty in community-dwelling older adults aged 65 years and above. A total of 171 participants were included, and body composition, physical function, cognitive function, and questionnaire-based assessments were conducted. The findings suggest that phase angle (PhA) is associated with pre-frailty, particularly in older women. Because pre-frailty represents a transitional stage preceding frailty, early identification is essential. As PhA reflects nutritional status and cellular health, it may be a useful indicator of early functional decline in community settings.

As reported in previous studies, PhA declines with age and is associated with physical function [16], physical activity levels [17], and nutritional status [18]. It has also been recognized as an indicator of nutritional status and risk of malnutrition, with lower values reflecting impaired cellular integrity and disease-related malnutrition [19,20]. In the present study, PhA showed positive correlations with grip strength and knee extension strength, and weak correlations with TUG and maximum walking speed. These findings support the association between PhA and physical function and suggest its potential role in identifying pre-frailty.

ROC curve analysis identified age-adjusted cutoff values of 4.19° and 4.74° in older women, corresponding to points prioritizing sensitivity and specificity, respectively. However, the AUC was relatively low (approximately 0.65), indicating modest discriminative ability. Therefore, PhA alone may have limited utility as a standalone screening tool for pre-frailty and should be interpreted in combination with other clinical and functional indicators. Notably, these cutoff values are comparable to those reported in previous studies, including a PhA of 3.81° in women aged approximately 80 years [21] and a cutoff value of ≤4.35° for identifying the risk of requiring nursing care [11], supporting their potential clinical relevance.

PhA may also reflect body fluid distribution. In the present study, body fluid parameters such as ECW/TBW were measured but not included in the analysis. Previous studies have suggested that alterations in extracellular and intracellular water balance (e.g., ECW/ICW or ECW/TBW ratios) may influence PhA values. Therefore, changes in fluid distribution may partially explain the association between PhA and functional performance observed in this study, and this aspect should be further investigated in future research.

Sex differences were observed in the association between PhA and pre-frailty. While no significant association was found in men, PhA was significantly associated with pre-frailty in women. This may be explained by sex-specific differences in age-related changes in body composition, such as greater reductions in muscle mass and increases in body fat in women, which may be more strongly reflected in PhA. Previous studies have also reported lower PhA values in women [22,23]. The lack of a significant association in men may be due to the smaller sample size; therefore, further studies with larger male samples are needed. PhA measurement is simple, quick, and minimally invasive, making it suitable for community-based screening and preventive interventions. Bioelectrical impedance analysis devices are portable and may be useful for routine screening in community health programs, clinical settings, and long-term care facilities.

This study has several limitations. First, participants were recruited from only two regions in Fukushima Prefecture, which may limit generalizability. Second, the association between PhA and pre-frailty in men was not sufficiently examined due to the small sample size. Third, PhA values were obtained using the InBody S10 device, and measurements may vary across different bioelectrical impedance devices and manufacturers. Therefore, the cutoff values identified in this study should be interpreted as device-specific. Previous studies have also reported variability in phase angle measurements across different BIA devices [24,25,26]. In addition, although PhA is an important indicator, its diagnostic accuracy alone is limited. Given the modest AUC value (0.65), comprehensive screening approaches combining PhA with other indicators may be required. Future studies should include larger samples to establish sex-specific cutoff values and further explore associations with factors such as regional characteristics, lifestyle, and nutritional status. Longitudinal studies are also needed to evaluate the effectiveness of interventions based on PhA screening.

In conclusion, phase angle measured by bioelectrical impedance analysis may serve as a simple and non-invasive indicator for identifying pre-frailty in community-dwelling older adults, particularly when used as part of a comprehensive assessment.

## 5. Conclusions

This study demonstrated that phase angle (PhA) derived from bioelectrical impedance analysis is associated with pre-frailty in community-dwelling older adults. In older women, age-adjusted cutoff values of 4.19° and 4.74° were identified based on points prioritizing sensitivity and specificity, respectively. However, given the modest discriminative ability observed, PhA alone may have limited utility as a standalone screening tool. These findings suggest that PhA may serve as a practical, non-invasive indicator for identifying pre-frailty when used in combination with other clinical and functional measures.

## Figures and Tables

**Table 1:** Basic characteristics by sex of the study participants.

	All	Men	Women	*p*-Value
*n*	171	36	135	
Age (years)	74.9 ± 5.8	76.4 ± 7.1	74.5 ± 5.3	0.15
Height (cm)	154.5 ± 7.3	163.4 ± 5.2	152.1 ± 5.7	<0.05
Weight (kg)	54.5 ± 9.3	61.2 ± 9.9	52.7 ± 8.2	0.79
BMI (kg/m^2^)	22.8 ± 3.4	22.9 ± 3.1	22.8 ± 3.4	0.14
KCL score (points)	4.5 ± 3.6	4.1 ± 4.5	4.6 ± 3.3	0.48
MFS (points)	11.3 ± 3.0	11.4 ± 3.4	11.2 ± 2.9	0.83
MoCA-J (points)	24.5 ± 3.4	23.6 ± 2.6	24.7 ± 3.5	0.08
Knee extension strength (Nm/kg)	1.37 ± 0.39	1.39 ± 0.47	1.37 ± 0.37	0.76
Grip strength (kg)	25.8 ± 6.4	33.7 ± 6.3	23.7 ± 4.5	<0.05
One-leg stance time with eyes open (s)	35.0 ± 22.4	30.1 ± 23.1	36.3 ± 22.0	0.15
TUG (s)	7.1 ± 1.1	7.0 ± 1.1	7.1 ± 1.0	0.47
Maximum walking speed (m/s)	1.9 ± 0.3	2.0 ± 0.3	1.9 ± 0.3	0.44
Body composition: PhA (°)	4.7 (4.4–5.1)	5.0 (4.6–5.4)	4.6 (4.3–5.0)	<0.05
Body composition: SMI (kg/m^2^)	6.3 (5.7–6.8)	7.4 (6.7–7.9)	6.1 (5.6–6.5)	<0.05

Data for all variables are presented as the mean ± standard deviation (SD), except for body composition analysis, which is shown as the median (interquartile range). BMI, body mass index; KCL, Kihon checklist; MFS; motor fitness scale; MoCA-J, Japanese version of Montreal cognitive assessment; TUG, timed up and go, PhA, phase angle; SMI, skeletal muscle index.

**Table 2:** Sex-related comparison of pre-frail/frail and robust groups subdivided based on KCL scores.

	Men			Women		
	Pre-Frail/Frail (KCL Score ≥ 4)	Robust (KCL Score 0–3)	*p*-Value	Pre-Frail/Frail (KCL Score ≥ 4)	Robust (KCL Score 0–3)	*p*-Value
*n*	15	21		67	68	
Age (years)	78.1 ± 7.7	75.1 ± 6.4	0.24	75.4 ± 5.4	73.5 ± 5.0	<0.05
Height (cm)	162.9 ± 5.7	163.8 ± 4.9	0.62	151.1 ± 5.5	153.2 ± 5.7	<0.05
Weight (kg)	60.0 ± 9.6	62.1 ± 10.1	0.53	52.1 ± 9.0	53.2 ± 7.4	0.45
BMI (kg/m^2^)	22.6 ± 3.2	23.1 ± 3.0	0.64	22.8 ± 3.6	22.7 ± 3.3	0.45
KCL score (points)	7.9 ± 4.6	1.4 ± 1.1	<0.05	7.2 ± 2.5	1.9 ± 1.1	<0.05
MFS (points)	9.5 ± 4.3	12.7 ± 1.4	<0.05	10.1 ± 3.1	12.3 ± 2.2	<0.05
MoCA-J (points)	22.8 ± 2.9	24.2 ± 2.1	0.11	24.1 ± 3.7	25.4 ± 3.3	<0.05
Knee extension strength (Nm/kg)	1.40 ± 0.32	1.74 ± 0.40	<0.05	1.21 ± 0.30	1.40 ± 0.39	<0.05
Grip strength (kg)	31.6 ± 7.4	35.1 ± 4.9	0.11	23.0 ± 4.4	24.5 ± 4.6	0.06
One-leg stance time with eyes open (s)	15.3 ± 15.5	40.7 ± 21.8	<0.05	34.5 ± 22.3	38.0 ± 21.5	0.35
TUG (s)	7.3 ± 1.1	6.8 ± 1.1	0.15	7.4 ± 1.1	6.9 ± 0.9	<0.05
Maximum walking speed (m/s)	1.9 ± 0.3	2.0 ± 0.3	0.15	1.9 ± 0.3	2.0 ± 0.3	<0.05
Body composition: PhA (°)	4.6 (4.3–5.4)	5.1 (4.9–5.4)	0.25	4.6 (4.3–4.8)	4.8 (4.4–5.1)	<0.05
Body composition: SMI (kg/m^2^)	6.7 (6.2–7.6)	7.5 (7.0–8.0)	0.18	5.9 (5.6–6.4)	6.3 (5.7–6.5)	0.61

Data for all variables are presented as the mean ± standard deviation (SD), except for body composition analysis, which is shown as the median (interquartile range). BMI, body mass index; KCL, Kihon checklist; MFS; motor fitness scale; MoCA-J, Japanese version of Montreal cognitive assessment; TUG, timed up and go, PhA, phase angle; SMI, skeletal muscle index.

**Table 3:** Correlations between PhA and physical function measures.

Variable	MFS (r)	Grip Strength (r)	Knee Extension Strength (r)	Maximum Walking Speed (r)	TUG (r)
PhA	0.36 **	0.54 **	0.16 *	0.20 *	−0.17 *

Notes: Values are presented as correlation coefficients (r). ** *p* < 0.01, * *p* < 0.05. PhA, phase angle; MFS, Motor Fitness Scale; TUG, Timed Up and Go.

**Table 4:** Multivariate logistic regression analysis of factors associated with pre-frailty in older women.

Variable	OR (95% CI)	*p*-Value
Phase angle (per 1° increase)	2.38 (1.08–5.23)	<0.05
Age (per 1-year increase)	0.96 (0.89–1.03)	0.22

OR, odds ratio; CI, confidence interval. The dependent variable was pre-frailty (yes/no). The model was adjusted for phase angle and age.

## Data Availability

The datasets generated and/or analyzed during the current study are not publicly available, but are available from the corresponding author upon reasonable request.
